# Recent Advances in Polymer-Based Biosensors for Food Safety Detection

**DOI:** 10.3390/polym15153253

**Published:** 2023-07-30

**Authors:** Binhui Wang, Da Huang, Zuquan Weng

**Affiliations:** 1 College of Biological Science and Engineering, Fuzhou University, Fuzhou 350108, China; binhwng@163.com; 2 Ministry of Education Key Laboratory for Analytical Science of Food Safety and Biology, Fujian Provincial Key Laboratory of Analysis and Detection for Food Safety, College of Chemistry, Fuzhou University, Fuzhou 350108, China

**Keywords:** polymer, food contaminants, food safety, biosensors

## Abstract

The excessive use of pesticides and drugs, coupled with environmental pollution, has resulted in the persistence of contaminants on food. These pollutants tend to accumulate in humans through the food chain, posing a significant threat to human health. Therefore, it is crucial to develop rapid, low-cost, portable, and on-site biosensors for detecting food contaminants. Among various biosensors, polymer-based biosensors have emerged as promising probes for detection of food contaminants in recent years, due to their various functions such as target binding, enrichment, and simple signal reading. This paper aims to discuss the characteristics of five types of food pollutants—heavy metals, pesticide residues, pathogenic bacteria, allergens, and antibiotics—and their adverse effects on human health. Additionally, this paper focuses on the principle of polymer-based biosensors and their latest applications in detecting these five types of food contaminants in actual food samples. Furthermore, this review briefly examines the future prospects and challenges of biosensors for food safety detection. The insights provided in this review will facilitate the development of biosensors for food safety detection.

## 1. Introduction

In recent years, food safety issues have become a major concern globally. The widespread utilization of pesticides has resulted in soil pollution, which has subsequently led to the detection of pesticide residues in numerous agricultural products, including those consumed by humans. Furthermore, these residues have even been found in human blood samples [1]. Food allergies are a significant public health burden in developed countries, drawing considerable attention. Notably, Australia has emerged with the highest prevalence of food allergies among developed nations, reaching an alarming rate of 10%. In contrast, other developed countries observe an incidence varying from 1% to 5% [2]. Pathogenic microorganisms have the potential to degrade seafood, leading to its spoilage. In the unfortunate event of consuming spoiled seafood, when these pathogenic microorganisms enter the body, they can pose significant food safety risks [3]. The presence of heavy metals not only hampers the growth and compromises the quality of crops but also leads to their accumulation within crops, ultimately finding their way into the human body through the food chain. Furthermore, it is worth noting that most heavy metals have been linked to genetic damage and an elevated risk of cancer development [4]. Therefore, it is essential to assess the quality of food to protect human health. Pesticide residues, illegal additives, allergens, pathogenic microorganisms, heavy metals, herbicides, and other risk factors present in food can significantly threaten human health [5]. Given the direct impact of food safety on human health, consumers are increasingly vigilant about the safety of the food they consume [6]. Therefore, the detection of food contaminants is of utmost importance. Inspectors analyze food for certain contaminants and allergens to determine whether they comply with local laws and regulations [7]. Traditional detection methods such as mass spectroscopy (MS), high-performance liquid chromatography (HPLC), enzyme-linked immunosorbent assay (ELISA), and gas chromatography (GC) have relatively high sensitivity and selectivity. However, these methods require expensive equipment and experienced operators, and the detection process is time-consuming, making them unsuitable for widespread use [8]. Thus, there is a critical need to develop methods that allow simple, fast, convenient, and highly sensitive detection of food contaminants [9].

Biosensors are analytical devices that are sensitive to biological substances and convert their concentrations into optical or electrical signals for detection. They are widely used to detect chemical substances containing biological components, such as enzymes, antibodies, and microorganisms [10]. Biosensors play a crucial role in various fields, including drug development, food processing, food safety testing, and environmental pollution detection [11]. Typically, biosensors are designed to perform rapid tests and generate a detectable signal when the molecular probe in the detector interacts with the desired analyte. This characteristic makes biosensors valuable tools for detecting a broad range of analytes, with high sensitivity and specificity [12]. Biosensors can be classified into several types, including electrochemical, piezoelectric, optical, mechanical, and thermal sensors. This categorization is based on the specific divisions of transducer types utilized in biosensing applications [13]. Sensors used for food detection are generally electrochemical biosensors and optical biosensors. The principle of electrochemical biosensors is based on the measurement of electrical signals generated by biochemical reactions occurring at the sensing interface. These biosensors typically consist of three main components: a recognition element (such as enzymes, antibodies, or DNA probes) that selectively interacts with the target analyte, a transducer that converts the biochemical signal into an electrical signal, and an electronic system for signal processing and analysis. The electrochemical biosensor operates by detecting changes in electrical properties, such as current or potential, resulting from the biochemical reaction between the recognition element and the target analyte. This interaction leads to the generation of a measurable electrical signal, which can be correlated with the concentration of the analyte of interest [14,15]. Optical biosensors typically consist of three main components: a recognition element (such as antibodies, enzymes, or nucleic acids) that selectively binds to the target analyte, a transducer that converts the biochemical signal into an optical signal, and a detector that measures the optical signal and provides the corresponding output. The principle of optical biosensors is based on the detection and analysis of changes in light signals resulting from the interaction between a biological recognition element and a target analyte. These biosensors utilize the properties of light, such as absorption, reflection, fluorescence, or refraction, to quantitatively or qualitatively measure the presence and concentration of specific analytes [16,17].

In the past decades, a variety of materials including metal nanoparticles, inorganic nanoparticles, quantum dots, and polymers have been employed to develop biosensors for on-site detection of food contaminants. Among them, polymers have shown great potential as candidates for developing biosensors that can be useful for food safety detection because polymers have many advantages for biosensing applications, including their ability to be easily functionalized with different biological or chemical sensing elements and their ability to be easily fabricated into a variety of shapes and sizes [18]. Polymers can be used as the sensing material in different types of biosensors, including optical, electrochemical, and mechanical biosensors [19]. In optical biosensors, polymers can be used as a matrix material for immobilizing fluorescent dyes or other chromophores that change their optical properties in response to the presence of a target analyte. In electrochemical biosensors, polymers can be used as the sensing material for detecting the electrochemical changes that occur when a target analyte interacts with the sensing material. Conductive polymers possess remarkable electrical conductivity, making them highly advantageous for augmenting the sensitivity of biosensors. In addition, specific types of conductive polymers demonstrate the unique ability to self-heal, consequently prolonging the operational lifespan of the sensor [20]. In mechanical biosensors, polymers can be used as the sensing material for detecting changes in the mechanical properties of the polymer when it interacts with a target analyte. In conjunction, polymer-based hydrogels offer commendable flexibility, thereby mitigating the potential for skin irritation [21]. Therefore, polymer-based biosensors have been widely used for a wide range of applications, including medical diagnosis, environmental monitoring, and food safety testing.

**Figure 1:**
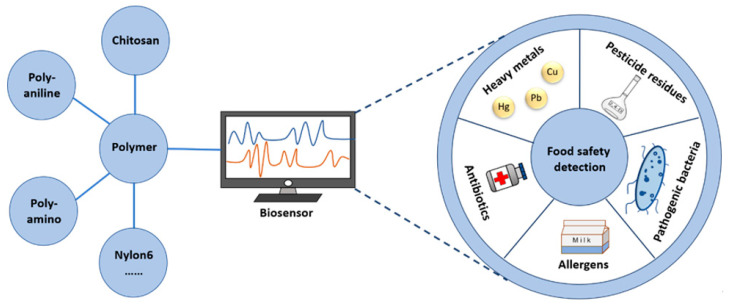
A schematic overview of polymer-based biosensors for food safety detection of different contaminants.

Herein, we present an overview of the recent advances in the field of polymer-based biosensors for food safety testing. Specifically, we focus on the detection of five categories of contaminants, namely heavy metals, pesticide residues, pathogenic bacteria, allergens, and antibiotics. We also discuss the detection principles of these biosensors and their applications in detecting food contaminants (Figure 1).

## 2. Polymer-Based Biosensors for Detection of Heavy Metals

Heavy metals are present in humans in extremely low levels. However, an increase in the concentration of heavy metals in the body can pose a serious threat to human life. Unfortunately, due to escalating environmental and industrial pollution, heavy metals have become ubiquitous in daily food such as vegetables, fruits, and water. Their consumption can lead to severe health problems and can even induce cancer, renal dysfunction, immune system imbalance, and other debilitating conditions [22]. Hence, it is imperative to detect and monitor the levels of heavy metals in food. Herein, we summarized the applications of polymer-based biosensors in detection of heavy metals (see Table 1 below).

**Figure 2:**
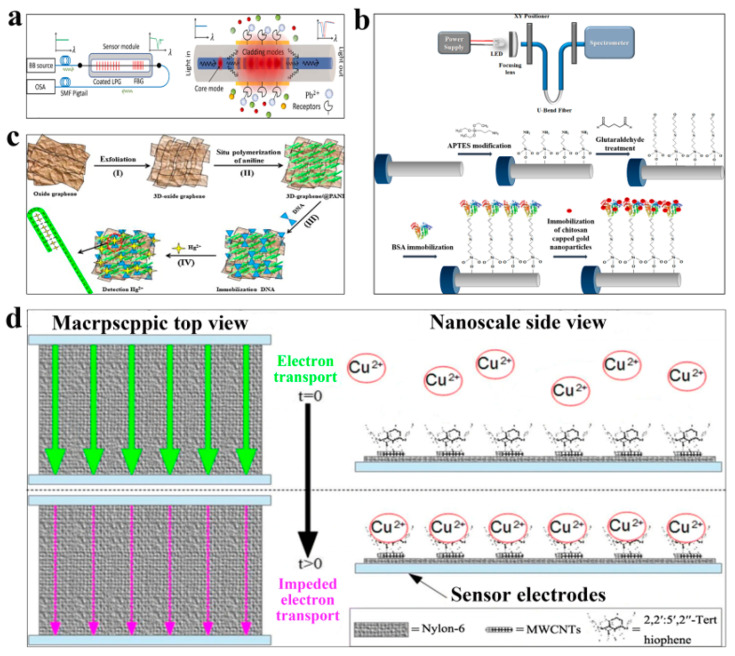
Polymer-based biosensors for detection of heavy metals. (**a**) Heavy metal ion biosensor based on LPG and FBG for detection of Pb^2+^. Reprinted with permission from reference [23], copyright Elsevier 2022. (**b**) Optical fiber biosensor based on chitosan coated with gold nanoparticles for detection of Hg^2+^. Reprinted with permission from reference [25], copyright Elsevier 2019. (**c**) Electrochemical biosensor based on polyaniline for detection of Hg^2+^. Reprinted with permission from reference [26], copyright Elsevier 2015. (**d**) Optical fiber current biosensor based on nylon 6 nanofibers for detection of Cu^2+^. Reprinted with permission from reference [30], copyright Springer 2019.

Lead (Pb) is one of the most common heavy metals, which can easily cause central nervous system damage in the human body, such as affecting human behavior, hearing impairment, etc. In severe cases, it may cause brain damage. Pb also interferes with the form of heme, leading to anemia [31]. Ghosh et al. employed long-period grating (LPG) and fiber Bragg grating (FBG) to create a fiber biosensor for heavy metal ion detection [23]. Graphene oxide, cross-linked chitosan composite, and poly-propionic acid were coated on the surface of the optical fiber to give the biosensor a better selectivity for Pb^2+^ detection. The attenuation bands of LPG and FBG were optimized to adapt to the optical S-band (Figure 2a). The biosensor can detect Pb^2+^ through the selective adsorption of heavy metals, which can realize the interaction between the high-order cladding mode fading field and the surrounding refraction. Deshmukh et al. modified polypyrrole/single-walled carbon nanotubes, synthesized by electrochemical means, with ethylenediaminetetraacetic acid to create a nanocomposite platform for biosensors [24]. The biosensor can detect Pb^2+^ ions with high sensitivity, with a detection limit of 0.07 μM.

Mercury (Hg) is another common heavy metal, which can also easily accumulate in the human body and threaten human life and health. High levels of Hg in the human body can cause damage to the brain, heart, lungs, and other organs [32]. Therefore, it is very important to detect the content of Hg in food. Sadani et al. developed an optical biosensor based on localized surface plasmon resonance [25]. The biosensor immobilizes chitosan-coated gold nanoparticles onto fiber functionalized with bovine serum albumin (Figure 2b) for detecting Hg^2+^ ions. The biosensor exhibited high sensitivity, with a detection limit of 0.1 ppb for tap water. Yang et al. synthesized a three-dimensional reduced graphene oxide and polyaniline (3DrGO@PANI) by oxidative polymerization, using DNA as the adsorbent for detecting Hg^2+^ ions in aqueous solutions (Figure 2c) [26]. The biosensor exhibits high selectivity for Hg^2+^ ions in the range of 0.1 nM to 100 nM, with a detection limit of 0.035 nM. Raril et al. modified the graphene electrochemical biosensor by poly-glycine and tested Hg^2+^ through voltammetry cycling technology [27]. The biosensor also demonstrates high selectivity, stability, and sensitivity.

The accumulation of other heavy metals such as copper (Cu), chromium (Cr), and zinc (Zn) in the human body can also cause damage to the human body. Excess Cu may lead to neurological diseases such as Alzheimer’s disease [33]. A large amount of Cr in the human body will also have bad effects, which may cause human lung cancer and mitochondrial damage [34]. Small amounts of Zn are important for the body, boosting DNA and protein synthesis, but too much Zn in the body can cause diarrhea and headaches [35]. Wong et al. utilized polyurethane-wrapped β-carotene to create a biosensor capable of detecting heavy metals, such as Al, Cu, Pb, and Zn [28]. The biosensor operates on the principle that the reaction between different heavy metals and β-carotene leads to a change in optical density values. Heavy metals can be detected by measuring and observing the change in optical density values at λ = 450 nm, using a simple spectrophotometric instrument. Alizadeh et al. developed an innovative sensing platform for the detection of chromium utilizing carbon composite electrodes [29]. In this study, the researchers synthesized Cr^3+^ molecularly imprinted polymer (MIP) nanoparticles by employing methylene succinic acid as the monomer and ethylene glycol dimethacrylate as the crosslinker. Remarkably, the biosensor exhibits a low detection limit of 17.6 n mol/L for Cr^3+^ without the requirement of converting it to metallic Cr. Lu et al. have developed a biosensor to detect Cu^2+^ in drinking water using fabric materials coated with nylon 6 nanofibers, multi-walled carbon nanotubes, and 2,2′:5′,2″-terthiophene molecules [30]. The biosensor operates on the principle of current obstruction, generated by the adsorption of Cu^2+^ ions onto the biosensor (Figure 2d). Remarkably, the biosensor can detect Cu^2+^ ions in the range of 0.65–39 ppm, exhibiting the advantages of sensitivity, flexibility, and portability.

## 3. Polymer-Based Biosensors for Detection of Pesticide Residues

Modern agriculture has long relied on the use of pesticides to mitigate the damage caused by insect pests, fungi, weeds, and other virus-borne diseases, ultimately enhancing crop yields. Nevertheless, excessive use of pesticides can leave behind harmful pesticide residues in farm products. These residues not only compromise the nutritional value of agricultural products but also pose a serious threat to human health. The impact of pesticide residues is not limited to acute poisoning but also includes potential damage to the nervous, respiratory, and reproductive systems, liver damage, and carcinogenic, teratogenic, and mutagenic chronic poisoning, which can have long-term effects on the human body [36]. Furthermore, the use of pesticides contributes to long-term pollution of water and soil, which may significantly impact future generations [37]. Therefore, there is an urgent need to develop highly selective and sensitive methods to detect pesticide residues in agricultural products. In this part, we present the applications of polymer-based biosensors in detection of pesticide residues (see Table 2 below).

**Figure 3:**
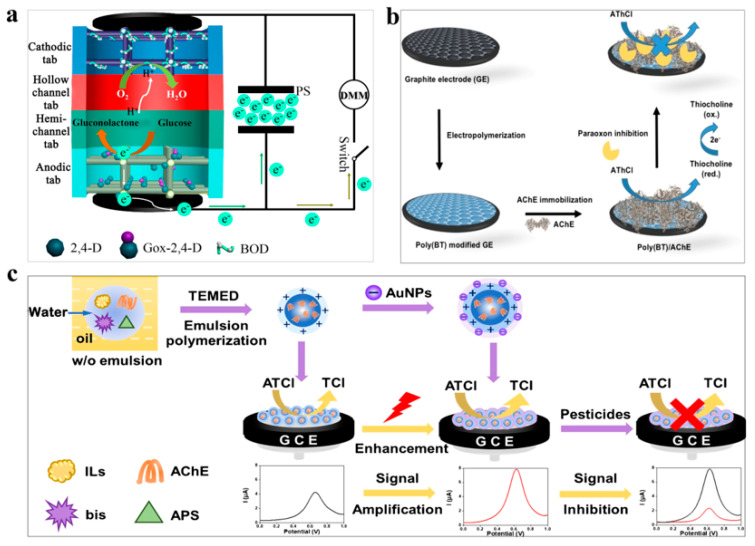
Polymer-based biosensors for detection of pesticide residues. (**a**) Self-powered biosensor based on MIP. Reprinted with permission from reference [38], Multidisciplinary Digital Publishing Institute 2022 for detection of 2.4-D. (**b**) Biosensor based on the BT polymer for detection of organophosphorus. Reprinted with permission from reference [40], copyright Elsevier 2022. (**c**) Electrochemical biosensor based on PILS for detection of carbaryl and dichlorvos. Reprinted with permission from reference [42], copyright Springer 2022.

The herbicide 2,4-dichlorophenoxyacetic acid (2,4-D) is commonly used in agriculture to eliminate weeds. Due to its stability, it tends to persist on crops and pose a threat to human health by interfering with the endocrine system. Hence, rapid and sensitive detection of 2,4-D before crops are sold or processed is important for protecting the consumers. Wang et al. developed a self-powered biosensor for detecting 2,4-D [38]. In this biosensor, glucose oxidase (GOx) and bilirubin oxidase (BOD) were utilized as the bioanode and biocathode, respectively, while MIP were grafted to the anode for specific recognition of 2,4-D. The resulting current was stored in a paper supercapacitor, amplified, and output by a digital multimeter (DMM) (Figure 3a), enabling self-powered sensing and providing a novel idea for the design and development of point-of-care testing equipment. In another study, Sok et al. developed a biosensor by fixing carbon nano-onions in a cyclodextrin substrate and depositing the synthesized nano-composite material on a screen-printing electrode [39]. The presence of 2,4-D could be detected through cyclic voltammetry, as it inhibits horseradish peroxidase and responds to the current. The biosensor was able to detect 2,4-D at concentrations as low as 0.023 μM. These biosensors provide highly selective and sensitive methods for detecting pesticide residues in agricultural products, thus addressing the urgent need to reduce the negative impact of pesticides on human health and the environment.

Organophosphorus pesticides are known inhibitors of acetylcholinesterase (AChE), which can lead to the accumulation of acetylcholine in the body and result in damage to human nerve function [51]. To prevent food safety issues aroused by organophosphorus pesticides residues in agriculture products, several biosensors have been developed for sensitive and rapid detection of these pesticides. Celik et al. developed an electrochemical biosensor for the measurement of organophosphorus compounds [40]. The biosensor employed a poly-2,2′-(9,9-dioctyl-9H-fluorene-2,7-diyl) bistiophene (BT) polymer that was synthesized and deposited onto a graphite electrode surface. The purpose of the polymer deposition was to facilitate the immobilization of the AChE on the electrode surface. AChE enzymes were utilized as targets for organophosphorus compounds, enabling the detection of such compounds. (Figure 3b). Notably, the biosensor exhibited a detection limit of 0.033 μg/L for acetylcholine chloride. Additionally, the biosensor was successfully applied for the detection of organophosphorus compounds in milk and drinking water samples. Lakshmi et al. utilized chitosan to functionalize Au@CeO_2_ synthesized by wet chemical synthesis, forming a film on an indium tin oxide electrode and fixing nucleic acid aptamers to detect chlorpyrifos with a lower limit of 0.12 pM [41]. Wan et al. designed an electrochemical detection platform based on sandwich core-shell nanocomposites [42]. In the composite, the AChE was encapsulated in the polymeric ionic liquids as the core, and the Au nanoparticles were assembled in the outer layer (Figure 3c). The detection limits of carbaryl and dichlorvos were 5.0 × 10^−2^ ng/mL and 3.9 × 10^−2^ ng/mL in this biosensor, respectively, with high sensitivity. Silva et al. synthesized poly-(brilliant cresyl blue) films on a glassy carbon electrode modified by carbon nanotubes through an electropolymerization reaction and developed a biosensor for detecting dichlorvos with a detection limit of 1.6 nm [43]. In addition, Zhou et al. constructed a biosensor platform by depositing phaseoloidin doped poly (3,4-ethyloxythiophene) (PL/PEDOT) composite onto the surface of a glass carbon electrode and fixing the poly amidoamine polymers on the PL/PEDOT, and single-stranded DNA was used as the aptamer to detect acetamiprid [44]. The acetamiprid concentration can be measured by the variation of the differential pulse voltammetry signal and the variation of the chronoamperometry signal. Combining the advantages of PEDOT, PL, and poly amidoamine polymers, the obtained biosensor had good conductivity and biocompatibility, and has been successfully used for the detection of acetamiprid in cabbage. Zhang et al. proposed an electrochemical biosensor based on chondroitin sulfate and polypyrrole for the detection of acetamiprid, in which acetamiprid aptamers were fixed on polypyrrole nanowires synthesized by chondroitin sulfate and polypyrrole [45]. After incubating with acetamiprid, its electrochemical concentration would change, allowing for the detection of acetamiprid with high sensitivity. These biosensors provide an effective way to detect organophosphorus pesticides and can be used in a variety of settings to improve public health.

Alphacypermethrin is a neurotoxic pyrethroid insecticide used to control pests in fruits and vegetables. Bhandari et al. developed a composite membrane consisting of chitosan and silver nanowires, which was used to modify Pt and detect alphacypermethrin using cyclic voltammetry. This biosensor exhibits high sensitivity for the detection of alphacypermethrin [46]. Isoproturon is a phenyl urea herbicide that targets annual weeds and is known for its slow degradation and accumulation in the food chain, which can be harmful to humans. Mehtab et al. developed a new polymer nanocomposite consisting of polymethylmethacrylate and ferrite for use in a modified glass carbon electrode to detect isoproturon [47]. This biosensor showed a good current response. Carboxin is a pesticide that inhibits the growth of crop-damaging bacteria by targeting their mitochondrial enzyme succinate dehydrogenase. Hasanjani et al. developed a biosensor to detect carboxin by modifying a pencil graphite electrode with a composite of DNA/poly L-methionine-silver nanoparticles [48]. The sensitivity of the modified composite pencil graphite electrode was significantly increased, and the detection limit was 5.0 pM.

Phenolic compounds are commonly used as biopreservatives for improving the shelf life of perishable products in food and agriculture industries, but they can be toxic to both humans and the environment. According to the United States Environmental Protection Agency (US EPA), phenolic compounds have been classified as potentially toxic chemicals due to their ability to readily dissolve in aquatic environments with minimal degradability [52,53]. These compounds have the potential to enter the human body through skin contact or ingestion and are associated with various health risks including central nervous system and respiratory system disturbances, as well as skin irritation [54]. Yasa et al. constructed a biosensor for the detection of catechol by fixing laccase on the electrode and coating it with thienopyrrole-based conjugated polymer and carbon dots on the graphite electrode to improve its response. The biosensor had a detection limit of 1.23 μM [49]. Erkmen et al. developed a biosensor platform for the detection of catechol using iridium oxide, poly(3,4-ethylenedioxythiophene) nanocomposite, and tyrosinase. The biosensor showed a detection limit of 0.017 μM for catechol [50].

## 4. Polymer-Based Biosensors for Detection of Pathogenic Bacteria

Bacterial infections have emerged as a significant global challenge, posing a substantial threat to human health and food safety. Several pathogenic bacteria including *Escherichia coli* (*E. coli*), *Mycobacterium tuberculosis* (*M. tuberculosis*), and *Staphylococcus aureus* (*S. aureus*), among others, are recognized as major contributors to this issue [55]. Despite the use of sterilization techniques, such as pasteurization and ultra-high temperature treatment, to eliminate bacteria in food, foodborne bacteria continue to be a primary cause of illness in both developed and developing countries. Thus, the development of effective biosensors for detecting pathogenic bacteria in food is of utmost importance [56]. In this section, we summarized the applications of polymer-based biosensors in detection of pathogenic bacteria (see Table 3 below).

**Figure 4:**
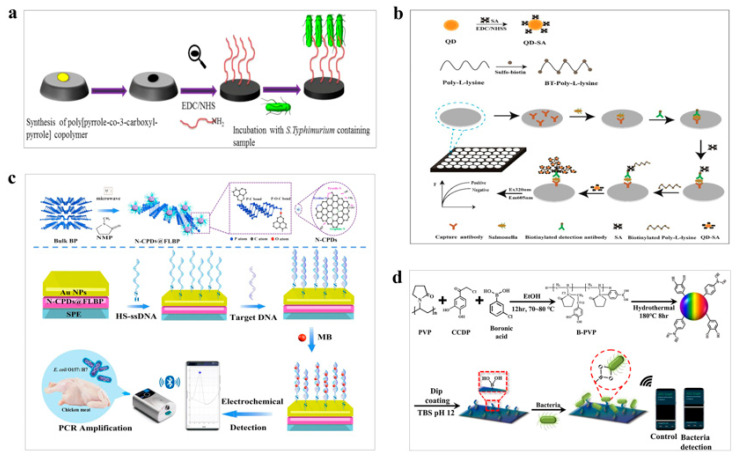
Polymer-based biosensors for detection of pathogenic bacteria. (**a**) Electrochemical biosensor based on pyrrole-co-3-carboxyl-pyrrole for detection of *S. typhimurium*. Reprinted with permission from reference [57], copyright Springer 2022. (**b**) Fluorescent biosensors based on poly-amino polymer for detection of *S. typhimurium*. Reprinted with permission from reference [58], copyright Elsevier 2022. (**c**) Biosensor based on N-CPDs-modified gold nanoparticles for detection of *E. coli*. Reprinted with permission from reference [60], copyright Elsevier 2022. (**d**) Biosensors based on B-PD for detection of *S. aureus*. Reprinted with permission from reference [62], copyright Elsevier 2021.

*Salmonella typhimurium* (*S. typhimurium*) is a common cause of acute enteritis, characterized by gastrointestinal symptoms such as diarrhea, fever, nausea, and vomiting [66]. In order to improve the accuracy and speed of *S. typhimurium* detection, various biosensors have been developed by researchers. Sheikhzadeh et al. developed a biosensor for the detection of *S. typhimurium* by combining polymers and aptamers [57]. The biosensor utilizes a pyrrole-co-3-carboxyl-pyrrole polymer film as its foundation. The carboxyl group present on the polymer film facilitates the immobilization of an amino-modified aptamer. The detection of *S. typhimurium* is achieved by monitoring the alterations in the electrical properties of the copolymer caused by the binding of *S. typhimurium* to the aptamer (Figure 4a). The biosensor exhibits a detection limit of 3 CFU/mL for *S. typhimurium*, enabling rapid and efficient detection of this bacterium in food samples. Liu et al. utilized poly-amino polymer poly-L-lysine as the probe skeleton and water-soluble quantum dots as a fluorescent probe to detect *S. typhimurium* by the streptomycin-biotin system [58]. Biotin-modified poly-lysine was used to amplify the fluorescence signal (Figure 4b). The biosensor accurately and quickly detected *S. typhimurium* with a detection limit of 4.9 × 10^3^ CFU/mL. Hu et al. assembled quantum dots layer by layer on the surface of polymer nanospheres, which were based on carboxylated poly(styrene/acrylamide) copolymer, to prepare nanorods and used them as signal reporting molecules for lateral flow immunoassay [59]. The nanorods had stronger fluorescence intensity, resulting in the detection of two fluorescence lines, test line and control line, under the UV lamp in the presence of *S. typhimurium*. The biosensor showed good specificity and selectivity. These biosensors demonstrate promising potential for *S. typhimurium* detection and could greatly improve diagnostic accuracy and speed in clinical and food safety settings.

*E. coli* is a globally significant foodborne pathogen that has been associated with numerous outbreaks of hemorrhagic colitis [67]. To address this issue, several researchers have developed miniature biosensors that can detect *E. coli* with high sensitivity and specificity. Shi et al. built a miniature biosensor for detecting *E. coli* using a single piece of silicon integrated on the chip [60]. The biosensor was composed of gold nanoparticles modified by N-methyl-2-pyrro-lidone carbonized polymer dots (N-CPDs) and methylene blue as an indicator. The biosensor employed single-probe DNA for immobilization (Figure 4c), which enabled the detection of *E. coli* in chicken. This biosensor demonstrated a detection limit of 3.33 × 10^−20^ mol/L, and possessed the characteristics of high specificity, high sensitivity, and good repeatability. Ren et al. employed lipopolysaccharides with specificity towards gram-negative bacteria, and used pulse induction of polypyrrole nanoarray, to generate slight scale deformation for amplifying the detection signal [61]. This biosensor for *E. coli* exhibited a detection limit of 1 CFU/mL, with the characteristics of being fast and portable, and having broad application prospects in portable equipment. Jo et al. coated the electrode with polymer dots, based with polyvinyl pyrrolidone, modified by boric acid for the detection of bacteria [62]. The biosensor allowed coating on the electrode surface due to the presence of catechol, and boric acid modification provides selectivity for bacterial capture. After trapping the bacteria, the biosensor detected the bacteria through the change of conductivity (Figure 4d). The biosensor could be combined with wireless sensing devices, and demonstrated high sensitivity for detecting both gram-positive and gram-negative bacteria. The minimum detection limit was 10^0.8^ CFU/mL for *E. coli* and 10^1^ CFU/mL for *S. aureus*. Fatema et al. fabricated unmarked biosensors based on graphene-polymer nanocomposites and nanopore materials [63]. The biosensor relied on the conductivity balance of polymer channels after the interaction of mesoporous and graphene-polymer to detect *E. coli*, and demonstrated high stability throughout the resistivity measurement process. Overall, these studies represent significant contributions to the development of biosensors for *E. coli* detection, each utilizing unique strategies and materials to achieve high sensitivity and specificity. These advancements hold promise for future applications in food safety and public health.

*Pseudomonas aeruginosa* (*P. aeruginosa*) is a bacterium that typically presents with low pathogenicity, but can cause infections in immunocompromised individuals leading to multiple lesions in different parts of the body, including otitis media, pneumonia, urinary tract inflammation, and blood infections [68]. Zhong et al. developed a fluorescent biosensor probe using polydopamine-polyethyleneimine which was synthesized by self-polymerization of dopamine than cross-linking with branched polyethyleneimine [64]. The use of a double gamete instead of a single aptamer significantly improved the detection sensitivity of the biosensor. Moreover, the biosensor’s feasibility was confirmed by detecting *P. aeruginosa* in food samples. In a separate investigation, Sarabaegi et al. devised a biosensor utilizing MIP and aptamers for the detection of *P. aeruginosa* [65]. The biosensor employed gold nanoparticles deposited onto a glassy carbon electrode as a substrate for the immobilization of the aptamer. The MIP integration was accomplished by voltammetric polymerization of dopamine onto the electrode surface. Gold nanoparticles were employed as intermediates in this biosensor to enhance the loading efficiency of aptamer sequences. Remarkably, the biosensor demonstrated a detection limit of 1 CFU/mL for *P. aeruginosa*, thus facilitating sensitive detection of this bacterium.

## 5. Polymer-Based Biosensors for Detection of Allergens

Food allergens are components of food that can trigger abnormal immune responses in the body. Food allergy is a complex condition that exhibits significant individual variability and is strongly linked to the strength of an individual’s immune system [69]. While mild food allergies can manifest as swelling of the lips or face, hives, tracheal constriction or twitching, vomiting, abdominal pain, or diarrhea, severe cases can be life-threatening, and in some instances, fatal. It is important to note that not all foods contain allergens, and among those that do, only a few are responsible for the majority of allergic reactions. Allergens are often shared among foods within the same family, particularly among plant foods. For example, individuals with a peanut allergy may also have varying degrees of allergic reactions to other legumes. The most common allergens are found in crustaceans, eggs, milk and dairy products, and peanuts [70]. Therefore, it is critical to be able to detect allergens in food. In this section, the applications of polymer-based biosensors in detection of allergens have been summarized in Table 4 below.

Shellfish allergy is one of the most common food allergies and a significant public health issue. Tropomyosin (TM), a heat-stable protein that is difficult to degrade or inactivate under high temperatures, is the primary allergen in shellfish and poses a considerable health risk to individuals with shellfish allergies [80]. Mohamad et al. developed a nanocomposite interface by combining Pd nanoparticles with polyaniline on a screen-printed carbon microelectrode [71]. This interface exhibits excellent conductivity and serves as a highly sensitive electrochemical biosensor. Notably, the biosensor demonstrates an impressive detection limit of 0.01 pg/mL for TM, making it suitable for the precise detection of TM content in seafood samples. Furthermore, the biosensor exhibits remarkable stability and reproducibility, enhancing its reliability for repeated detection. Another study by Wang et al. reported the development of a biosensor based on an aptamer detection probe [72]. They constructed a poly-dopamine layer on Fe_3_O_4_ to allow the biosensor to obtain a recognition probe directly bound to TM. The presence of magnetic Fe_3_O_4_ enables quick separation of the analyte when it is bound to the probe, which greatly enhances the detection efficiency. Additionally, the probe was modified with peptides to prevent probe contamination. The researchers also linked a probe to carbon quantum dots and constructed the above two probes into a biosensor. This biosensor had a detection limit of 30.76 ng/mL and exhibited both high specificity for TM and high accuracy. Overall, these biosensors offer promising approaches to detecting shellfish allergens and addressing the significant health risk they pose to individuals with shellfish allergies.

**Figure 5:**
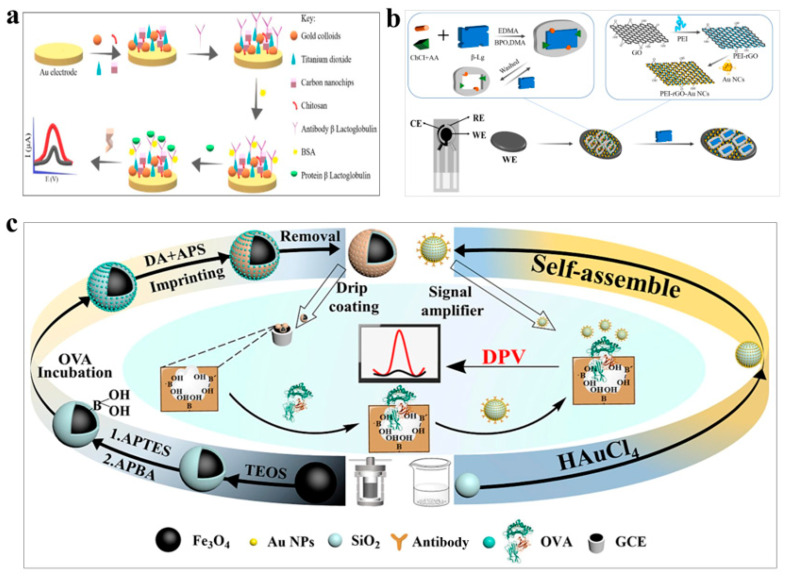
Polymer-based biosensors for detection of allergens. (**a**) Electrochemical biosensors based on polymer chitosan for detection of β-LG. Reprinted with permission from reference [73], copyright Wiley 2021. (**b**) Biosensors based on quantum dot aptamers for detection of β-LG. Reprinted with permission from reference [74], copyright Elsevier 2023. (**c**) Electrochemical biosensor based on OVA sandwich-structured MIP for detection of ovalbumin. Reprinted with permission from reference [77], copyright Elsevier 2023.

β-lactoglobulin (β-LG) is a major milk allergen that can cause a range of allergic reactions, including skin irritation, respiratory distress, gastrointestinal issues, and even systemic allergic reactions [81]. To detect β-LG, several biosensors have been developed that employ innovative techniques to detect the allergen in real food samples [82]. Hong et al. developed an electrochemical biosensor that uses the conductive polymer chitosan to detect β-LG [73]. In this biosensor, TiO_2_ and carbon nano-chips nanocomposites were immobilized in gold electrodes and then inoculated with β-LG antibody. The presence of β-LG leads to the binding of the allergen to the antibodies and generates an electrical signal that allows for the detection of β-LG (Figure 5a). The biosensor had a detection limit of 0.01 pg/mL and demonstrated excellent performance in detecting β-LG in real food samples. Wang et al. developed an electrochemical biosensor for the detection of β-lactoglobulin by modifying electrodes with MIP [74]. The MIP were synthesized using β-LG as a template and subsequently immobilized on nanocomposites composed of polyethyleneimine, reduced graphene oxide, and gold nanoclusters (Figure 5b). The biosensor exhibited excellent responsiveness to β-LG, with a remarkable detection limit of 10^−9^ mg/mL. The biosensor platform’s detection limit for β-LG was 0.048 mg/L, which was consistent with commercial kits, and demonstrated good practicality. Amor-Gutierrez et al. designed a biosensor by utilizing poly-lysine-modified graphite-wire-printed electrodes and using methylene blue-labeled anti-β-LG as an aptamer [75]. When β-LG was present, the aptamer underwent a conformational change, leading to a change in the methylene blue conformation with the electrode surface, enabling β-LG detection. The biosensor had a detection limit of 0.09 ng/mL and was effective in detecting β-LG in real food samples. In summary, these biosensing techniques offer innovative and sensitive approaches to detecting β-LG in real food samples, enabling better management of milk allergies.

Ovalbumin, also known as albumin, is a glycoprotein that contains trace amounts of phosphorus and is a high-quality protein [83]. However, some individuals, particularly infants, are susceptible to allergic reactions that can result in itching, diarrhea, and other symptoms, which can be fatal if left untreated [84]. Therefore, biosensors have been developed for the detection of ovalbumin, utilizing innovative techniques that offer high sensitivity and selectivity. Mohamad et al. developed a biosensor for the detection of ovalbumin utilizing the conductive polymer chitosan [76]. Pd nanoparticles were synthesized and bound to Fe_3_O_4_, then dispersed in chitosan to form nanocomposites, which were then cast onto graphene electrodes, grafted with 4-aminobenzoic acid, and conjugated with ovalbumin antibody to the electrode surface. The biosensor detected ovalbumin through the synergistic conductive interaction between nanocomposites and chitosan, with a detection limit of 0.01 pg/mL for ovalbumin. Fu et al. developed a sandwich-structured molecular imprinted electrochemical biosensor for accurate measurement of ovalbumin [77]. The detection principle involved dropping the imprinted layer onto the molecular-imprinted polymer and inserting it into the ovalbumin solution to rebond it. The non-electrically active protein blocked the imprinted cavity, preventing transfer between the electrode and the solution, and amplifying the signal using SiO_2_ nanoparticles (Figure 5c). The detection limit of the sensor was 3.0 fg/mL, demonstrating excellent selectivity and sensitivity for the detection of ovalbumin. In summary, these biosensing techniques offer innovative and sensitive approaches to detecting ovalbumin, enabling better management of allergic reactions associated with this protein. The development of such biosensors has the potential to improve the diagnosis and management of ovalbumin allergy and improve the health outcomes of affected individuals.

Peanut allergy is a prevalent and serious health issue, for which there is currently no antidote. Prevention of peanut consumption is the only known treatment, making the detection of peanut allergens crucial in the food industry [85]. The most common allergenic proteins in peanuts are Ara h1, Ara h2, and Ara h3, while Ara h6 and Ara h2 are potential allergens. In recent research, two biosensors have been developed for detecting Ara h1 and Ara h2 [86]. Jiang et al. created a non-contact sensing platform by immobilizing rat basophilic mast cells in a composite gel consisting of polyvinyl alcohol, gelatin methacryloyl, and nano-hydroxyapatite, integrated with paper fibers [78]. Real-time detection of Ara h2 was achieved by measuring changes in capacitance, where the presence of the allergen led to a significant reduction in capacitance. This biosensor had a detection limit of 0.028 ng/mL and exhibited greater utility than conventional cell sensors. Similarly, Sun et al. developed a biosensor for detecting Ara h1 by coating a composite of chitosan and carbon nanotubes on glass carbon electrodes and plating a gold film on the composite through electrodeposition [79]. Detection of Ara h1 was accomplished by monitoring electrochemical reduction of DNA hybridization, allowing the separation of biotin, streptavidin, and horseradish peroxidase from the electrode in the presence of target DNA. This biosensor had a detection limit of 0.013 fmol/L and showed promise for the clinical diagnosis and food safety of peanut allergens. These novel electrochemical DNA biosensors have the potential to revolutionize the detection of peanut allergens in the food industry and clinical settings, ultimately leading to improved prevention and treatment of peanut allergies.

## 6. Polymer-Based Biosensors for Detection of Antibiotics

Antibiotics are a group of secondary metabolites produced by microorganisms, animals, and plants that exhibit anti-pathogenic properties or other biological activities. They are known to interfere with the vital functions of living cells. In animal husbandry, antibiotics are frequently utilized to improve growth rates and combat bacterial infections [87]. However, the excessive and inappropriate use of antibiotics can result in their accumulation in animal products such as meat and milk. If such contaminated food is consumed by humans, it can lead to bacterial resistance, decreased immunity, and severe allergic reactions, posing a significant threat to human health [88]. Consequently, the development of biosensors for detecting antibiotics is essential. In recent years, various biosensors have been designed and implemented for detecting antibiotics. These biosensors are highly sensitive, accurate, and efficient, and they can detect antibiotics at low concentrations. They operate on diverse principles such as fluorescence, electrochemistry, and nanotechnology, and they can be used to detect a wide range of antibiotics. Biosensors can help to ensure the safety of food products and prevent the overuse of antibiotics, ultimately safeguarding human health. Herein, the applications of polymer-based biosensors in detection of antibiotics have been demonstrated in Table 5 below.

**Figure 6:**
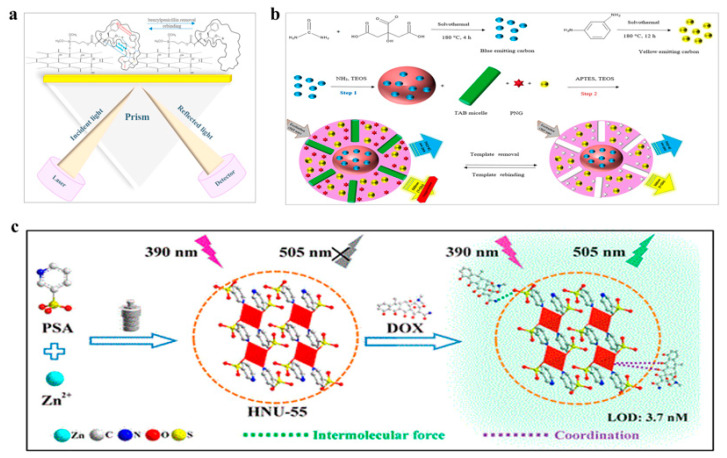
Polymer-based biosensors for detection of antibiotics. (**a**) Biosensor based on penicillin-imprinted polymers for detection of benzylpenicillin. Reprinted with permission from reference [89], copyright Elsevier 2023. (**b**) Fluorescent biosensor based on silica microsphere for detection of benzylpenicillin. Reprinted with permission from reference [90], copyright Elsevier 2020. (**c**) Biosensor based on HNU-55 for detection of Dox. Reprinted with permission from reference [96], copyright Amer Chemical Soc 2021.

Benzylpenicillin is a widely used antibiotic, but its excessive use in livestock to prevent and treat diseases can result in residues in milk, meat, and kidneys, posing a risk to food safety [98]. Celik et al. developed a biosensor based on penicillin-imprinted polymers (Figure 6a) [89]. The biosensor employed an optical surface plasmon resonance (SPR) sensor functionalized with a hydrophobic N-methacryloyl-L-phenylalanine methyl ester monomer for imprinting. The biosensor demonstrated high selectivity for benzylpenicillin, being 8.16 and 14.04 times more selective than amoxicillin and ampicillin, respectively. The biosensor was tested for benzylpenicillin detection in both aqueous solutions and milk samples and exhibited good sensitivity and specificity. Another study by Jalili et al. involved doping blue emissive carbon dots into silica microspheres and preparing yellow-emissive carbon dots on the surface of microspheres by sol-gel method, which were embedded in a polymer layer (Figure 6b) [90]. The composite displayed two emission peaks that could be used as response and reference signals at excitation wavelengths of 560 nm and 440 nm. In the presence of benzylpenicillin, the fluorescent color changed from yellow to blue, allowing for the detection of benzylpenicillin. The biosensor had a detection limit of 0.34 nM and excellent selectivity and recognition, and could be detected by the naked eye, making it highly promising for practical applications. Hu et al. developed a sol-gel-based biosensor for the detection of benzylpenicillin [91]. The biosensor was prepared by coating a mixture of Fe_3_O_4_, carbon nanotubes, and chitosan onto the surface of a carbon electrode. Additionally, SiO_2_ was applied to the surface of the nanoparticles to enhance the biosensor’s stability. Remarkably, the biosensor demonstrated a low detection limit of 1.5 × 10^−9^ mol/L for benzylpenicillin samples.

Chloramphenicol (CAP) is a widely used antibiotic in the treatment of animal diseases due to its efficacy and low cost. However, the accumulation of excessive amounts of CAP in the body can cause aplastic anemia, which can be fatal if left untreated [99]. Consequently, various biosensors have been developed to tackle this concern by enabling the detection of CAP. Barveen et al. developed a biosensor for CAP detection based on gold nanostar-enhanced Raman signals [92]. Gold nanostars were synthesized directly on a poly(methyl methacrylate) film, and the resulting hotspots generated an enhancement of Raman signals when the light was reduced to the poly(methyl methacrylate). The biosensor was used to detect CAP in chicken wing samples and exhibited good mechanical stability and reproducibility, with a detection limit of 3.67 × 10^−9^ M. Shaheen et al. used graphitic carbon nitride nanomaterials and MIP to develop a biosensor [93]. They fabricated a nanomaterial and an assembly of MIP by polymerizing free radicals to form a nanointerface, with CAP serving as the template analyte. The nanointerface was used as a mass-sensitive biosensor to detect CAP, and the addition of the analyte reduced the fundamental frequency of the device, resulting in a sensor signal. The biosensor demonstrated a detection specificity of 90 to 98%. Chu et al. prepared molecularly imprinted polyaniline nanotubes for CAP detection by synthesizing polyaniline nanoparticles on a gold electrode using chronoamperometry and cyclic voltammetry [94]. The electrochemical process of polyaniline on the electrode surface was thoroughly investigated to enable the detection of CAP. Notably, the biosensor exhibited a remarkable detection limit of 1.24 × 10^−9^ M in water plant culture samples. One of the notable advantages of this biosensor is its ease of fabrication and cost-effectiveness. Roushani et al. coated 3-aminomethyl pyridine functionalized graphene oxide on the surface of glass carbon electrode and then coated silver nanoparticles on the above materials [95]. The CAP complex was attached to the composite, and when the CAP was removed, the MIP cavity cooperated with the cavity embedded in the aptamer to construct the nanohybrid receptor. Due to the dual accuracy of molecular imprinting and aptamer, the biosensor had excellent sensing performance with a detection limit of 0.3 pm for CAP, and it was suitable for the detection of milk samples.

Doxycycline (Dox) is a commonly used broad-spectrum antimicrobial agent to treat bacterial infections in poultry and aquaculture [100]. However, excessive use of Dox can lead to its accumulation in fish and subsequent transfer to humans through the food chain. This accumulation can cause adverse gastrointestinal reactions, such as nausea, vomiting, abdominal pain, and diarrhea [101]. Sun et al. developed a novel biosensor for the detection of Dox [96]. The biosensor is based on a polymer, HNU-55, synthesized using Zn^2+^ and 3-pyridinesulfonic acid. The polymer can bind to Dox, and when illuminated with light at 390 nm, it emits fluorescence, enabling easy detection of Dox (Figure 6c). The biosensor is highly selective and exhibits ultra-high sensitivity. The luminescence studies of the system indicate that the luminescence intensity of HNU-55 is linearly proportional to the concentration of Dox, with an extremely low detection limit of 3.7 nM. Ashley et al. synthesized a new composite material for the detection of Dox [97]. The composite material is composed of magnetic cores and fluorescent MIP. The MIP exhibit strong specificity for Dox and can recognize it efficiently. The composite material uses the fluorescence quenching effect of Dox and fluorescein for easy detection. The biosensor shows significant Dox-dependent fluorescence quenching in an aqueous environment. It demonstrates good linearity in the range of 0.2 to 6 µM with a detection limit of 117 nM. Overall, these biosensors offer promising tools for the detection of Dox. The use of these biosensors can provide a sensitive, selective, and rapid means of monitoring Dox levels in the environment and the food chain, which can ultimately help to reduce the health risks associated with its excessive use.

## 7. Conclusions and Perspectives

This review focuses on polymer-based biosensors and their applications in detecting heavy metals, pesticide residues, pathogenic bacteria, allergens, and antibiotics, five major categories of food contaminants. The biosensors’ operating principles, detection methods, and their applications in testing food samples such as water, milk, and chicken wings are discussed in detail. The biosensors employ various sensing principles, including nucleic acid hybridization, antigen-antibody binding, aptamer and target binding, enzyme activity inhibition, and electrochemical activity. The results of biosensor testing can be read through a variety of methods, including naked eye observation, Raman spectroscopy, voltammetry, photocurrent variation, and fluorescence spectroscopy.

Polymer-based biosensors offer greater flexibility than the sophisticated detection methods typically used in laboratories, as they can be employed in a variety of environments and detected on various media, such as thin films, fibers, and solutions. Nevertheless, polymer-based biosensors possess certain limitations, including complexity of sample preparation, cost considerations, interference and selectivity challenges, and limited stability. For example, many biosensors are bonded to electrodes through polymers, with the binding forces often being physical interactions. Such weak physical interaction forces may compromise the sensor’s stability, which makes it imperative that bonding strength of polymer materials to the electrode surface is improved to enhance the biosensor’s stability. Polymeric biosensors often face challenges in achieving high sensitivity and selectivity for target analytes. Strategies to enhance sensitivity can include optimizing the surface-to-volume ratio of the sensing element, designing polymer structures with tailored properties for specific analytes, or integrating signal amplification techniques. Improving selectivity can be achieved through the incorporation of selective receptors or functional groups into the polymer matrix. To enable widespread adoption, biosensors must be manufactured on a large scale. However, many polymeric materials and fabrication techniques may not be readily scalable. Researchers can explore scalable fabrication methods such as roll-to-roll printing, microfluidics, or 3D printing to overcome this limitation. Consequently, there remain a number of challenges that must be overcome before polymer-based biosensors can be used on a large scale or enter the market. Nonetheless, it is widely believed that the continued technological advancements in polymer science and nanotechnology will alleviate these limitations. As a result, novel polymer-based biosensors with enhanced performances such as rapid and on-site detection, multiplex detection capabilities, enhanced sensitivity and selectivity, integration with data analytics, and smart packaging and quality monitoring are expected to emerge for food safety detection.

Future biosensors may incorporate multiple sensing elements or functionalities within a single device, allowing simultaneous detection of multiple analytes or integration with other technologies like wireless communication or data processing systems. Once these advancements are achieved, the superiority of polymer-based biosensors will be fully displayed and they can further improve human health and living standards.

## Figures and Tables

**Table 1:** Polymer-based biosensors for detection of heavy metals.

Category	Polymeric Material	Biosensor Type	Limit of Detection	Linear Range	Reference
Pb	Chitosan	Optical biosensor	0.5 nM	nonlinear	[23]
Pb	Polypyrrole	Electrochemical biosensor	0.07 µM	0.15–800 µM	[24]
Hg	Chitosan	Optical biosensor	0.1 ppb	0.1–540 ppb	[25]
Hg	Polyaniline	Electrochemical biosensor	0.035 nM	0.1–100 nM	[26]
Hg	Poly-glycine	Electrochemical biosensor	6.6 µM	1.5 × 10^−4^–1.5 × 10^−3^ M	[27]
Al Cu Pb Zn	Polyurethane	Optical biosensor	-	0.10–10.00 ppm 0.10–10.00 ppm 0.01–10.00 ppm 0.01–1.00 ppm	[28]
Cr	Methylene succinic acid Crosslink ethylene glycol dimethacrylate	Electrochemical biosensor	17.6 nM	0.1–10 mmol/L	[29]
Cu	Nylon6	Electrochemical biosensor	0.65 ppm	0.65–39 ppm	[30]

**Table 2:** Polymer-based biosensors for detection of pesticide residues.

Category	Polymeric Material	Biosensor Type	Limit of Detection	Linear Range	Reference
2,4-D	Polypyrrole	Electrochemical biosensor	0.53 pM	1.0 pM–50.0 µM	[38]
2,4-D	Cyclodextrin	Electrochemical biosensor	0.023 μM	-	[39]
Organophosphorus	Poly-2,2′-(9,9-dioctyl-9H-fluorene-2,7-diyl) bistiophene	Electrochemical biosensor	0.033 μg/L	0.025–4 mM	[40]
Chlorpyrifos	Chitosan	Electrochemical biosensor	0.12 pM	10 pM–500 nM	[41]
Carbaryl Dichlorvos	Polymeric ionic liquid particles	Electrochemical biosensor	0.05 ng/mL 0.039 ng/mL	0.063–880 ng/mL 0.13–1400 ng/mL	[42]
Dichlorvos	Poly-brilliant cresyl blue	Electrochemical biosensor	1.6 nM	2.5–60 nM	[43]
Acetamiprid	Poly (3,4-ethyloxythiophene)	Electrochemical biosensor	0.0355 fg/mL	0.1–1 pg/mL	[44]
Acetamiprid	Polypyrrole	Electrochemical biosensor	0.065 fg/mL	1 fg/mL–0.1 ng/mL	[45]
Alphacypermethrin	Chitosan	Electrochemical biosensor	14 nM	10–100 nM	[46]
Isoproturon	Polymethylmethacrylate	Electrochemical biosensor	5 pM	6.5 × 10^−8^ M	[47]
Carboxin	Poly L-methionine	Electrochemical biosensor	5 pM	8 pM–1µM	[48]
Catechol	Poly-thienopyrrole	Electrochemical biosensor	1.23 μM	1.25–175 µM	[49]
Catechol	Poly(3,4-ethylenedioxythiophene)	Electrochemical biosensor	0.017 μM	0.05–10.65 µM	[50]

**Table 3:** Polymer-based biosensors for detection of pathogenic bacteria.

Category	Polymeric Material	Biosensor Type	Limit of Detection	Linear Range	Reference
*S. typhimurium*	Poly(pyrrole-co-3-carboxyl-pyrrole)	Electrochemical biosensor	3 CFU/mL	10^2^–10^8^ CFU/mL	[57]
*S. typhimurium*	Poly-L-lysine	Optical biosensor	4.9 × 10^3^ CFU/mL	4.9 × 10^3^–4.9 × 10^7^ CFU/mL	[58]
*S. typhimurium*	Poly(styrene/acrylamide)	Optical biosensor	5 × 10^3^ CFU/mL	5 × 10^4^–5 × 10^7^ CFU/mL	[59]
*E. coli*	N-methyl-2-pyrro-lidone carbonized polymer	Electrochemical biosensor	3.33 × 10^−20^ mol/L	1.0 × 10^−19^–1.0 × 10^–6^ mol/L	[60]
*E. coli*	Polypyrrole	Electrochemical biosensor	1 CFU/mL	1 × 10^0^–1 × 10^6^ CFU/mL	[61]
*E. coli*	Poly(polyvinyl pyrrolidone)	Electrochemical biosensor	10^0.8^ CFU/mL	10^1^–10^7^ CFU/mL	[62]
*E. coli*	Graphene–polymer	Electrochemical biosensor	1 CFU/μL	10^3^–10^5^ CFU/mL	[63]
*P. aeruginosa*	Polydopamine-polyethyleneimine	Optical biosensor	1 CFU/mL	10^1^–10^7^ CFU/mL	[64]
*P. aeruginosa*	Poly-dopamine	Electrochemical biosensor	1 CFU/mL	10^1^–10^7^ CFU/mL	[65]

**Table 4:** Polymer-based biosensors for detection of allergens.

Category	Polymeric Material	Biosensor Type	Limit of Detection	Linear Range	Reference
Tropomyosin	Polyaniline	Electrochemical biosensor	0.01 pg/mL	0.01–100 pg/mL	[71]
Tropomyosin	Poly-dopamine	Optical biosensor	30.76 ng/mL	0.1–2.5 μg/mL	[72]
β-lactoglobulin	Chitosan	Electrochemical biosensor	0.01 pg/mL	0.01–500 pg/mL	[73]
β-lactoglobulin	Polyethyleneimine	Electrochemical biosensor	10^−9^ mg/mL	10^−9^–10^−4^ mg/mL	[74]
β-lactoglobulin	Poly-lysine	Electrochemical biosensor	0.09 ng/mL	0.1–10 ng/mL	[75]
Ovalbumin	Chitosan	Electrochemical biosensor	0.01 pg/mL	0.01 pg/mL–1 µg/mL	[76]
Ovalbumin	Poly-dopamine	Electrochemical biosensor	3.0 fg/mL	1.0 × 10^−7^–1.0 × 10^–4^ mg/mL	[77]
Ara h2	Polyvinyl alcohol	Electrochemical biosensor	0.028 ng/mL	0.1–100 ng/mL	[78]
Ara h1	Chitosan	Electrochemical biosensor	0.013 fmol/L	3.91 × 10^−17^–1.25 × 10^–15^ mol/L	[79]

**Table 5:** Polymer-based biosensors for detection of antibiotics.

Category	Polymeric Material	Biosensor Type	Limit of Detection	Linear Range	Reference
Benzylpenicillin	Poly(hydroxyethyl methacrylate-graphene oxide-N-methacryloyl-L-phenylalanine)	Optical biosensor	0.021 ppb	1–100 ppb	[89]
Benzylpenicillin	Poly-tetraethoxysilane	Optical biosensor	0.34 nM	1–32 nM	[90]
Benzylpenicillin	Chitosan	Electrochemical biosensor	1.5 × 10^−9^ M	5.0 × 10^−8^–1.0 × 10^−3^ M	[91]
Chloramphenicol	Poly(methyl methacrylate)	Optical biosensor	3.67 × 10^−9^ M	10^−1^−10^−9^ M	[92]
Chloramphenicol	Poly-methacrylic	Optical biosensor	177 µM	-	[93]
Chloramphenicol	Poly-aniline	Electrochemical biosensor	1.24 × 10^−9^ M	10^−8^–10^−3^ M	[94]
Chloramphenicol	Resorcinol polymerization	Electrochemical biosensor	0.3 pM	1.0 pM–1.0 nM	[95]
Doxycycline	HNU-55	Optical biosensor	3.7 nM	0–30 μM	[96]
Doxycycline	Poly-methacrylic	Optical biosensor	117 nM	0.2–6 μM	[97]

## Data Availability

Not applicable.
